# Experimental Investigation of Eco-Friendly Anhydrous Calcium Sulfate Whisker and Waste Cooking Oil Compound Modified Asphalt Mixture

**DOI:** 10.3390/ma16062409

**Published:** 2023-03-17

**Authors:** Yutong Liu, Zeliang Yang, Hui Luo

**Affiliations:** School of Civil and Hydraulic Engineering, Huazhong University of Science and Technology, Wuhan 430074, China; liuyutong@hust.edu.cn (Y.L.);

**Keywords:** asphalt pavement, compound-modified asphalt mixture, environmentally friendly, anhydrous calcium sulfate whiskers (ACSW), waste cooking oil (WCO), performance

## Abstract

In recent years, waste material recycling and reuse have attracted great interest as environmentally friendly modifiers to improve asphalt pavement performance. In this study, anhydrous calcium sulfate whiskers (ACSW), synthesized using phosphogypsum waste, and waste cooking oil (WCO), one of the most prevalent waste oils, were used together as modifiers to create an environmentally friendly asphalt mixture. In particular, WCO was used to compensate for the negative effects of ACSW on asphalt mixture performance at low temperatures. A variety of ACSW and WCO compound-modified asphalt mixtures were fabricated. High-temperature stability, medium-temperature fatigue, low-temperature anti-cracking, moisture susceptibility, repeated freeze–thaw, and long-term aging tests were conducted to comprehensively evaluate the pavement performance. Compared to the base asphalt mixture, the compound-modified asphalt mixtures were demonstrated to have better high- and low-temperature, moisture susceptibility, fatigue, anti-freezing, and anti-aging properties, especially for the 6%ACSW and 2%WCO compound-modified asphalt mixture. Therefore, the 6%ACSW and 2%WCO compound-modified asphalt mixture was ultimately selected for use in construction, as this mixture can meet the requirements for regions with cold winters and hot summers.

## 1. Introduction

With the rapid expansion of highway transportation recently, asphalt pavement has been extensively applied in highways and urban roads because of its superior performance [1,2,3]. However, growing traffic volumes and loads, extreme climates, and degradation of traditional pavement materials affect asphalt mixture performance, leading to the early appearance of rutting, cracking, and spalling [4,5,6,7]. To enhance asphalt pavement performance while actively responding to the call for environmental protection and low-carbon energy conservation, waste resources are recycled and reused. Many studies have attempted to employ different solid waste materials to improve asphalt mixture quality, including waste rubber powder [8,9], crop straw [10,11], and wood waste [12,13].

Phosphogypsum is a type of solid waste generated during the production of phosphate fertilizer. Annual phosphogypsum generation is estimated to be 200–300 million tons globally [14,15,16,17]. Large volumes of phosphogypsum waste are dumped into the environment, polluting the soil and water and endangering human health. To minimize pollution and promote sustainable development, phosphogypsum waste could be utilized to synthesize anhydrous calcium sulfate whiskers (ACSW) [18,19,20], which are inorganic needle-like fibrous submicron materials. These materials offer high-temperature resistance, high toughness, chemical corrosion resistance, and high-cost performance [18,21]. ACSW are a promising modifier because of their desirable strength and modulus, which can improve certain properties of concrete and asphalt mixtures [22,23,24]. For example, a previous study showed that adding ACSW to asphalt mixtures improves the material’s high-temperature properties [25]. Li et al. [26] tested the high-temperature resistance to permanent deformation of an asphalt mixture modified with 4% ACSW. The findings indicated that ACSW created a three-dimensional interwoven mesh in the asphalt, improving permanent deformation resistance. Fan et al. [27] found that ACSW could significantly improve the Marshall stability of the asphalt mixture while reducing splitting tensile strength and failure strain when the content of ACSW was 10.8%. On the other hand, ACSW negatively affect the low-temperature properties of asphalt mixtures [28] due to the asphalt hardening produced by introducing ACSW, as well as the incompatibility of inorganic ACSW with organic asphalt binders, which are prone to fracturing under tensile stress at low temperatures. To overcome this disadvantage, compound-modified asphalt development has become increasingly important.

Waste cooking oil (WCO) refers to cooking waste generated in restaurants or households. Approximately five million tons of WCO are produced annually in China, and more than half of which is discarded, leading to adverse impacts on the environment and human health [29,30]. Only a small proportion of WCO is reused to produce biodiesel [31]. However, because WCO and asphalt binders are petroleum-based substances, WCO has a lower molecular weight and a high degree of unsaturation along with low viscosity and good flowability [32]. WCO can be employed as an alternative light oil to increase the flexibility of asphalt, thus improving the asphalt’s low-temperature properties [33,34]. Niu et al. [35] investigated the low-temperature properties of WCO-modified asphalt mixtures via indirect tensile and three-point bending tests. The authors found that when the WCO content was 5%, the low-temperature tensile strain could be enhanced by 90%. Yan et al. [36] tested the pavement behavior of WCO asphalt mixtures and observed that the presence of WCO not only improved low-temperature performance but also enhanced aging resistance. Yan et al. [37] found that the WCO-modified asphalt mixtures offered the best aging resistance when the WCO content was 6% based on Marshall stability and indirect tensile testing. The retained ratios of the mixtures were 98% and 107%, respectively, indicating that aging effects had less of an influence on the WCO-modified asphalt. Luo et al. [38] indicated that the reaction between WCO and asphalt was only physical, but the intensity of absorption peaks of some functional groups of asphalt was changed. This was due to increasing the light component content of the asphalt, which contributed to improving the low-temperature characteristics and construction compatibility of the asphalt. Due to its wide availability, WCO is effective in improving asphalt mixture performance, and its ability to be recycled promotes sustainable development. Therefore, WCO can be considered a modifier able to cooperate with ACSW to enhance asphalt mixture performance.

Based on the above factors, it is essential to investigate ACSW and WCO compound-modified asphalt mixtures. Although the physical and rheological characteristics of ACSW and WCO compound-modified asphalt binders have been explored by our laboratory, ACSW and WCO compound-modified asphalt mixtures remain uninvestigated. This work aims to evaluate different aspects of WCO and ACSW compound-modified asphalt mixture performance. The remaining sections of the study are organized as follows. Firstly, ACSW and WCO compound-modified asphalt mixtures with various proportions were fabricated. Then, high- and low-temperature, moisture susceptibility, medium-temperature fatigue, anti-freezing, and anti-aging performance were analyzed. The aim of this investigation is to thoroughly assess the performance of environmentally friendly compound-modified asphalt mixtures based on the performance benefits of ACSW and WCO. The results will serve as a reference for research on ACSW and WCO compound modification techniques and applications in the field of asphalt pavement materials.

## 2. Materials and Methods

### 2.1. Materials

The base asphalt binder (#70) was purchased from Wuhan Lubang Asphalt Pavement Engineering Co., Ltd. (Wuhan, Hubei, China), and the physical parameters are presented in Table 1. The parameters of the base asphalt binder met the specification requirements documented in JTG F40-2004 [39].

ACSW are inorganic needle-like fibrous submicron materials artificially synthesized from phosphogypsum waste. The surfaces of ACSW were treated with sodium carbonate and stearic acid to enhance their compatibility with the asphalt binder. The physical properties of treated ACSW are exhibited in Table 2.

WCO was collected from food garbage in Wuhan, Hubei Province, China. Waste fragments and crumbs of WCO were eliminated by simple filtering. The physical properties of WCO were demonstrated in Table 3.

The coarse aggregate, fine aggregate, and mineral filler employed in the test were limestone, and their physical parameters were assessed following JTG E42-2005 specifications [40]. The test results and requirements (JTG F40-2004 [39]) are exhibited in Table 4.

### 2.2. Preparation of Compound-Modified Asphalt Binder

**Figure 1:**
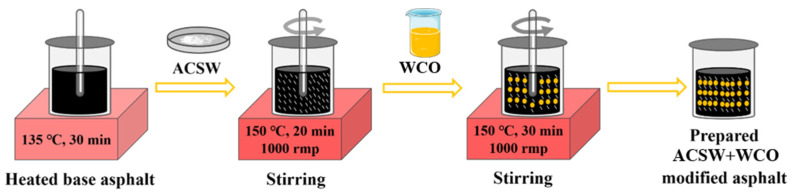
Flow chart of the compound-modified asphalt preparation.

According to the results of previous studies conducted in our laboratory on the physical and rheological behavior of ACSW and WCO compound-modified asphalt, we determined that 6%ACSW and 1%WCO, 6%ACSW and 2%WCO, 8%ACSW and 2%WCO compound-modified asphalts have the most desirable performance. Thus, the above ratios of ACSW and WCO compound-modified asphalt binders were produced, and ASCW single-modified asphalt was also produced as a control group. The preparation method for ACSW and WCO compound-modified asphalt was presented in Figure 1. First, the base asphalt binder was melted in an oven at about 135 °C, and then ACSW (based on the mass ratio of the base asphalt) were mixed with base asphalt and blended by a high-speed shear mixer, stirring at 1000 rpm for 20 min at 150 °C. Subsequently, a predetermined mass of WCO (based on the mass ratio of the base asphalt) was added and mixed for 30 min to guarantee uniform modifier distribution in the asphalt. Finally, using a thermostat, the mixed compound-modified asphalt binders were maintained at 150 °C for 30 min. To maintain the homogeneity of the test conditions, the base asphalt was processed in the same way. A total of 5 types of asphalt binders were designed to investigate asphalt mixture performance, and their abbreviations and physical properties are listed in Table 5.

### 2.3. Mix Design of Asphalt Mixtures

**Figure 2:**
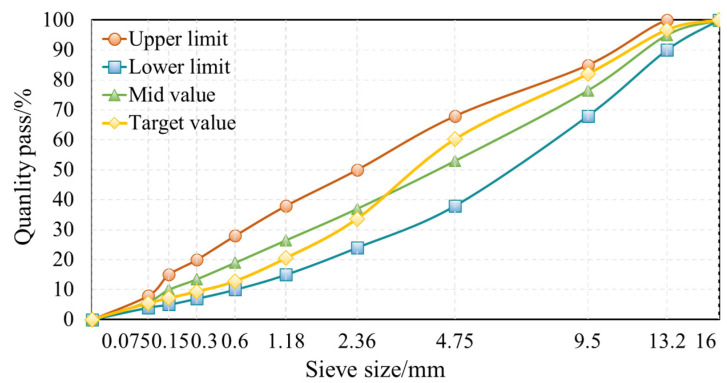
Synthetic gradation of aggregate (AC-13).

In this experiment, aggregate gradation of AC-13 was chosen to develop the behavior of the ACSW and WCO compound-modified asphalt mixture. The synthesized gradation of the aggregates is depicted in Figure 2. The optimum asphalt binder content (OAC) of different combinations of the ACSW and WCO compound-modified asphalt mixtures was evaluated by the Marshall test mixture design method (JTG F40-2004) [39]. The OAC and the mixture design parameters are exhibited in Table 6.

### 2.4. Experimental Methods

**Figure 3:**
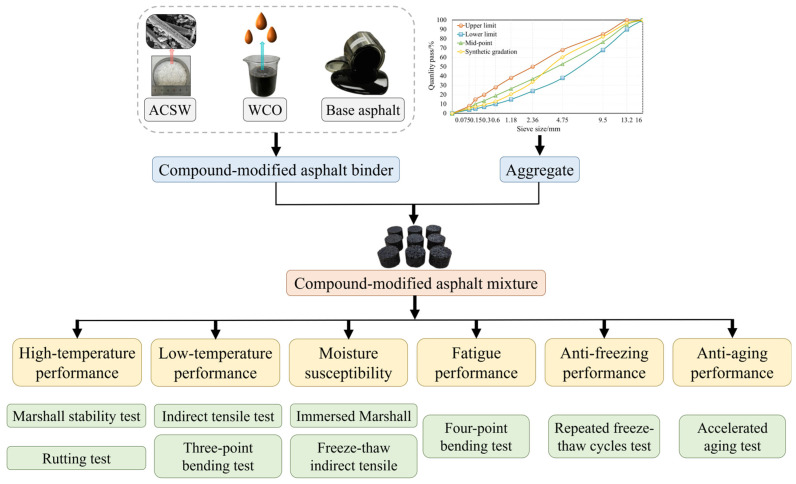
Materials and experimental processes.

In the investigation, the ACSW and WCO compound-modified asphalt mixture was assessed considering high-temperature, low-temperature, moisture susceptibility, fatigue, anti-freezing, and anti-aging performance. Three parallel tests were performed for each experimental test to reduce random errors and improve the accuracy of the experimental results. Figure 3 illustrates the testing materials and experimental processes.

#### 2.4.1. High-Temperature Performance Test

##### Marshall Stability Test

A Marshall stability test was performed according to the test method of T 0709, JTG E20-2011 [41]. First, the standard Marshall samples were placed in a 60 °C water bath and held for 35 min. Then, the samples were set and applied at a loading rate of 50 mm/min to obtain the Marshall stability (MS) and flow value (FL), and the Marshall modulus was measured to evaluate the high-temperature performance of the compound-modified asphalt mixtures, as shown in Equation (1):

(1) $$T=\frac{MS}{FL}$$

where T is the Marshall modulus (kN/mm), MS is the Marshall stability (kN), and FL is the flow value (mm).

##### Rutting Test

A rutting test was performed to investigate the resistance to permanent deformation of asphalt mixtures at high temperatures. According to the test method of T 0719, JTG E20-2011 [41], rutting plate samples 300 mm in length and width, and 50 mm in thickness were placed in a fixed temperature greenhouse at 60 °C for about 6 h. Then, the rutting test was performed at a contact pressure of 0.7 MPa and a rolling speed of 42 times/min. The rutting deformation at 45 min and 60 min was measured, and the dynamic stability (DS) was calculated to evaluate the high-temperature rutting resistance, as shown in Equation (2):

(2) $$DS=\frac{\left(T_{60}-T_{45}\right)\times N}{d_{60}-d_{45}}\times C_{1}\times C_{2}$$

where DS is dynamic stability (time/mm), $T_{60}$ and $T_{45}$ represent 60 min and 45 min, $d_{60}$ and $d_{45}$ represent the deformation of 60 min and 45 min, N is the rolling speed, and $C_{1}$ $C_{2}$ are the test machine and sample coefficient, both of which are 1.0.

#### 2.4.2. Low-Temperature Performance Test

##### Indirect Tensile Test

An indirect tensile strength test at −10 °C was conducted to assess the low-temperature anti-cracking of the compound-modified asphalt mixtures. According to the test method of T 0716, JTG E20-2011 [41], the Marshall specimens were maintained in a −10 °C air bath for at least 6 h. Following that, the specimens were loaded at 1 mm/min between two circular compression strips. The peak load and the relative deformation of the sample at the time of damage were measured. The indirect tensile strength (ITS) (Equation (3)), tensile strain (Equation (4)), and stiffness modulus (Equation (5)) were then calculated as follows:

(3) $$ITS=\frac{0.006287P}{h}$$

(4) $$\varepsilon =\frac{X\left(0.0307+0.0936\mu \right)}{1.35+5\mu }$$

(5) $$S=\frac{P\times \left(0.27+1.0\mu \right)}{h\times X}$$

where ITS is the indirect tensile strength (MPa), P is the peak load (N), h is the sample height (mm), ε is the tensile strain, X is the horizontal deformation (mm), μ is Poisson’s ratio of 0.25, and S is the stiffness modulus (MPa).

##### Three-Point Bending Test

A three-point bending test was performed to investigate the low-temperature tensile performance of the asphalt mixtures. Based on the test method of T 0715, JTG E20-2011 [41], asphalt mixture beam samples 250 mm (length), 30 mm (width), and 35 mm (height) were manufactured. The temperature of the test was −10 °C and the application rate was 50 mm/min. The peak load and mid-span flexure were measured at the moment of specimen damage, and the flexural tensile strength (Equation (6)) and flexural strain (Equation (7)) were calculated as follows:

(6) $$R=\frac{3\times L\times P}{2\times b\times h^{2}}$$

(7) $$\varepsilon =\frac{6\times h\times d}{L^{2}}$$

where R is the flexural tensile strength (MPa), P is the peak load (N), ε is the flexural strain (με), d is the mid-span flexure (mm), and L, b, and h are the length, width, and height of the specimen (mm).

#### 2.4.3. Moisture Susceptibility Test

##### Immersed Marshall Test

An immersion Marshall stability test was performed to assess the moisture susceptibility of the compound-modified asphalt mixtures that were subjected to water erosion at high temperatures. According to the test method of T 0709, JTG E20-2011 [41], one set of Marshall samples was held for 35 min in a 60 °C water bath, and the other set of Marshall samples was maintained for 48 h. Then, the test was performed separately under different conditions to calculate the immersion residual Marshall stability (IRMS), as shown in Equation (8):

(8) $$IRMS=\frac{MS_{immersion}}{MS}\times 100$$

where IRMS is the immersion residual Marshall stability (%), $MS_{immersion}$ is the stability of the specimen immersed for 48 h (kN), and MS is the stability of the standard Marshall test specimen (kN).

##### Freeze–Thaw Indirect Tensile Test

An indirect tensile test after the freeze–thaw was performed to test the moisture susceptibility of the asphalt mixtures at low temperatures. According to the test method of T 0729, JTG E20-2011 [41], one set of Marshall samples was kept at about 25 °C as a control group. The other set of Marshall samples was vacuum-filled with water first and then stored in a −18 °C refrigerator for 16 h, followed by a 60 °C bath for 24 h. After that, two sets of samples were maintained in a water bath for more than 2 h. The maximum load of the samples was measured, and the indirect tensile strength residual ratio (ITSR) after the freeze–thaw was calculated to assess moisture susceptibility, as shown in Equation (11):

(9) $$ITS_{c}=\frac{0.006287P_{c}}{h}$$

(10) $$ITS_{F-T}=\frac{0.006287P_{F-T}}{h}$$

(11) $$ITSR=\frac{ITS_{F-T}}{ITS_{c}}\times 100$$

where $ITS_{c}$ is the indirect tensile strength of the control set of samples (MPa), $P_{c}$ is the peak load of the control set of samples (N), h is the sample’s height (mm), $ITS_{F-T}$ is the indirect tensile strength of the samples undergoing freeze–thaw (MPa), and $P_{F-T}$ is the peak load of the samples undergoing freeze-thaw (N).

#### 2.4.4. Fatigue Performance Test

A four-point bending test was performed to assess the mid-temperature fatigue performance of the compound-modified asphalt mixtures. According to the test method of T 0739, JTG E20-2011 [41], similar to that of ASTM D7460 [42], asphalt mixture beam specimens that were 380 (length) × 65 (width) × 50 mm (height) were placed under 15 °C, with 10 Hz loading frequency and 1000 με strain control in the continuous bias sinusoidal loading mode. The normalized stiffness number product (*NM*) is one of the methods specified by ASTM D7460 for estimating fatigue life. In the relationship between *NM* and loading cycles, the loading times correspond to the peak value of the *NM* fatigue life, for which *NM* can be calculated using the following equation:

(12) $$NM=\frac{S_{i}\times N_{i}}{S_{0}\times N_{0}}$$

where $S_{i}$ is the stiffness modulus (Pa), $N_{i}$ is the loading times, $S_{0}$ is the stiffness modulus at the 50th loading (MPa), and $N_{0}$ refers to 50.

#### 2.4.5. Repeated Freeze–Thaw Cycles Test

The repeated freeze–thaw cycle test, compared to the moisture susceptibility test, can better reflect the degree of damage of asphalt mixtures in cold and frozen regions. This is because, in the freezing stage, the pore water of the asphalt mixture freezes, and freezing swelling occurs, while in the thawing stage, water flows into the new cracks produced by freezing and swelling. Thus, the repeated freezing and thawing effect will cause high- and low-temperature properties damage to the asphalt mixture. Referring to the moisture susceptibility test method, the repeated freeze–thaw cycle test process first involved vacuum-filling the chamber with water, followed by freezing at −18 °C for 16 h and then placing the mixture in 60 °C water for 8 h. The high-temperature Marshall stability and low-temperature indirect tensile strength were tested following 2, 4, 6, 8, and 10 repeated freeze–thaw cycles, and the loss rates (Equations (13) and (14)) were calculated to evaluate the anti-freezing performance:

(13) $$Loss_{MS}=\frac{MS_{0}-MS_{i}}{MS_{0}}\times 100$$

(14) $$Loss_{ITS}=\frac{ITS_{0}-ITS_{i}}{ITS_{0}}\times 100$$

where $Loss_{MS}$ is the Marshall stability loss rate (%), $MS_{0}$ is the Marshall stability of specimens without freeze–thaw cycles (kN), $MS_{i}$ is the Marshall stability of specimens after ith repeated freeze–thaw cycles (kN), $Loss_{ITS}$ is the loss rate of the indirect tensile strength (%), $ITS_{0}$ is the indirect tensile strength of specimens without freeze–thaw cycles (MPa), and $ITS_{i}$ is the indirect tensile strength of specimens after ith repeated freeze–thaw cycles (MPa).

#### 2.4.6. Anti-Aging Performance Test

The accelerated aging test simulated the effects of high temperature and air oxidation on the asphalt mixture’s pavement performance. According to the test method of T 0734, JTG E20-2011 [41], the short-term aging test was conducted first by heating the mixed asphalt mixture in a 135 °C oven for 4 h. Then, the long-term aging test was conducted by preparing Marshall specimens from the asphalt mixture that experienced short-term aging, followed by placing the mixture in an 85 °C oven for 120 h and then cooling it naturally for 16 h. After that, the high-temperature Marshall stability and low-temperature indirect tensile strength were tested. Finally, the aging Marshall stability ratio (AMSR) and aging indirect tensile strength ratio (AITSR) were calculated to evaluate the anti-aging effect:

(15) $$AMSR=\frac{MS_{A}}{MS}\times 100$$

(16) $$AITSR=\frac{ITS_{A}}{ITS}\times 100$$

where MS and $MS_{A}$ are the Marshall stability of specimens before and after aging, respectively, (kN), and ITS and $ITS_{A}$ are the indirect tensile strength values of specimens before and after aging (MPa).

## 3. Results and Discussion

### 3.1. High-Temperature Performance

#### 3.1.1. Marshall Stability

**Figure 4:**
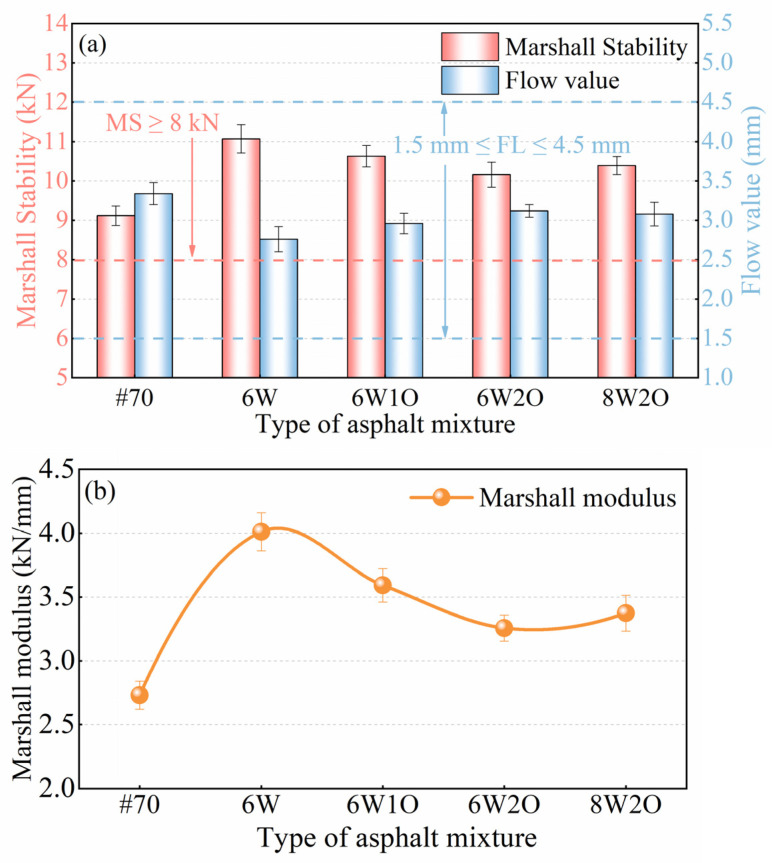
Test results of Marshall stability, flow value, and Marshall modulus. (**a**) Test results of Marshall Stability and flow value. (**b**) Test results of Marshall modulus.

The MS test results for the five types of asphalt mixtures are depicted in Figure 4a. We observed that the MS values of all types of asphalt mixtures were greater than 8 kN, thus meeting the specification criteria (JTG F40-2004) [39]. Due to the impact of ACSW and WCO, the MS of the modified asphalt mixtures were enhanced to some degree. Among them, the impact of the 6%ACSW single-modified mixture on the MS was the most significant, representing an increase from 9.12 to 11.07 kN (21.4%). After introducing WCO, it was found that the MS of the compound-modified asphalt mixtures gradually reduced with an increase in the WCO concentration. However, the MS of the 6%ACSW and 2%WCO compound-modified asphalt mixture was still 11.4% higher than that of the base asphalt mixture. Although WCO somewhat decreased the MS of the asphalt mixture, the high-temperature performance of the compound-modified asphalt mixture outperformed the base asphalt mixture when the content of ACSW and WCO was appropriate. Furthermore, when the ACSW proportion was enhanced to 8%, the MS increased by 2.3% compared to the 6%ACSW and 2%WCO compound-modified asphalt mixture. This result suggests that adding ACSW greatly enhanced the asphalt mixture’s load-bearing ability, possibly because the ACSW provided a three-dimensional interwoven mesh structure for the modified asphalt binder and influenced the arrangement of the asphalt’s molecular structure.

FL refers to the equivalent amount of deformation when the specimen’s Marshall stability achieves its highest value. The FL results of the asphalt mixtures are presented in Figure 4a. All values satisfied the demands of the technical standards for heavy traffic in long hot summer areas (i.e., an FL no less than 1.5 mm and no greater than 4 mm [39]). The FL of different types of modified asphalt mixtures were all increased. The change of the FL of the 6%ACSW modified mixture was the most significant, it decreased by 17.4% from 3.34 to 2.76 mm, indicating that adding ACSW can minimize the deformation of asphalt mixtures under loading. After adding WCO, the FL of the ACSW and WCO compound-modified asphalt mixture showed a gradual increase with an increase of WCO. However, the FL of the compound-modified asphalt mixtures was notably less than that of the base asphalt mixture.

The Marshall modulus is commonly used to reflect the force required for a unit deformation of the asphalt mixture to occur. The Marshall modulus results are illustrated in Figure 4b. The Marshall modulus of the modified asphalt mixtures were substantially greater than those of the base asphalt mixture. The influence of ACSW and WCO on the Marshall modulus was equivalent to that of MS. The Marshall modulus of the ACSW-modified asphalt mixture had the highest value of 4.01 kN/mm. Adding ACSW was able to dramatically enhance the Marshall modulus, thereby improving the deformation resistance at high temperatures. Adding WCO adversely affected the Marshall modulus to a certain degree, but compared to the base asphalt mixture, the Marshall modulus of the ACSW and WCO compound-modified asphalt mixtures showed a gradual increase. For compound-modified asphalt mixtures, it is not possible to focus on only one aspect of performance. Low-temperature anti-cracking, medium-temperature fatigue, moisture susceptibility, anti-freezing, and anti-aging performance will be discussed in the following sections.

#### 3.1.2. Dynamic Stability

**Figure 5:**
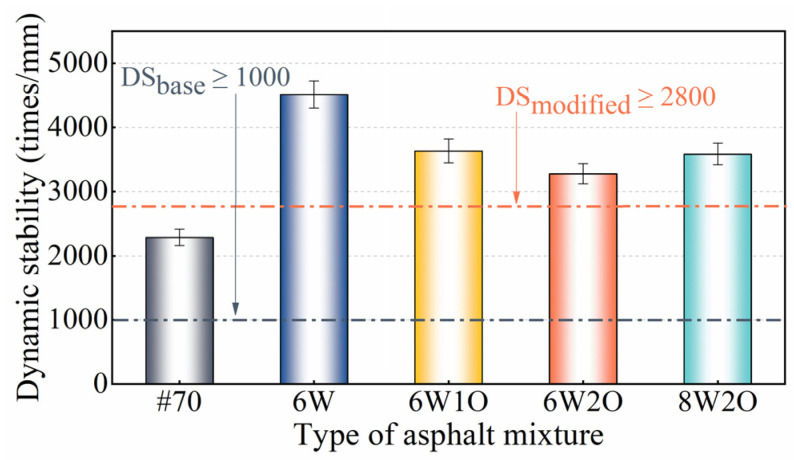
Test results of dynamic stability.

The DS is used here to indicate the asphalt mixture’s deformation resistance at high temperatures, as depicted in Figure 5. According to the specifications [39], the DS of the base asphalt mixture was 2287 times/mm, satisfying the limit of more than 1000 times/mm in hot summer regions, and the DS of all modified asphalt mixtures was more than 2800 times/mm, which also satisfied the corresponding requirement. In contrast to the base asphalt mixture, after the single addition of 6%ACSW, the DS of the modified asphalt mixture increased by 97.3% from 2287 to 4512 times/mm. This finding is consistent with the results obtained in an earlier investigation reported by Fan et al. [43] that the ACSW provide strength and stiffness to asphalt mixture, resulting in better-resisting deformation under high-temperature conditions. In contrast to the ACSW-modified asphalt mixture, there was a minor decrease in DS with an increase in the WCO level. However, in comparison to the base asphalt mixture, the DS of 6%ACSW and 1%WCO, 6%ACSW and 2%WCO, 8%ACSW and 2%WCO showed a significant increase of 58.9%, 43.4%, and 56.8%, respectively. Combining the findings of the Marshall and rutting tests, the ACSW and WCO compound modification showed an obvious enhancement in the asphalt mixture’s permanent deformation resistance under a high-temperature environment.

### 3.2. Low-Temperature Performance

#### 3.2.1. Anti-Cracking Performance

**Figure 6:**
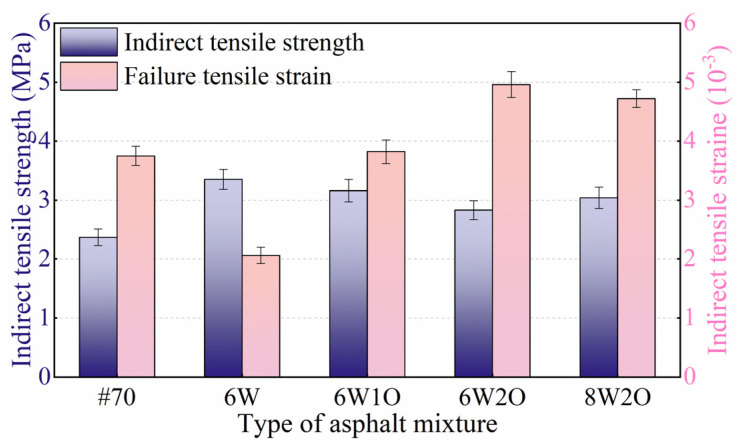
Test results of low-temperature indirect tensile strength and strain.

Higher ITS and higher failure tensile strain indicate greater low-temperature crack resistance in an asphalt mixture [36]. The low-temperature ITS test results are illustrated in Figure 6. The ITS of the modified asphalt mixtures were found to be larger than those of the base asphalt mixture. The asphalt mixture ITS increased from 2.37 to.3.35 MPa after the addition of 6%ACSW. This result shows that introducing ACSW obviously enhanced the low-temperature ITS. The ITS of the ACSW single-modified asphalt mixture was slightly reduced after the introduction of WCO. Notably, the ITS of the 6%ACSW and 1%WCO, 6%ACSW and 2%WCO, and 8%ACSW and 2%WCO compound-modified asphalt mixtures were still 33.3%, 19.4%, and 28.3% greater than that of the #70 asphalt mixture.

The failure tensile strain test results are presented in Figure 6. In contrast to the base asphalt mixture, the failure tensile strain decreased by 45.1% after adding 6%ACSW, which indicated that the ACSW modified asphalt mixture was prone to brittle fracture when subjected to a load at low temperatures. The failure tensile strain of the ACSW single-modified asphalt mixture increased obviously after incorporating WCO. When the WCO level increased to 2%, the failure tensile strain of the compound-modified asphalt mixture was found to be 32.3% larger than that of the base asphalt mixture, and also 2.4 times greater than that of the ACSW single-modified asphalt mixture. Continuing to increase the ACSW to 8%, it could be noted that the deformation ability of the 8%ACSW and 2%WCO compound-modified asphalt mixture was still better than that of the ACSW single-modified and base asphalt mixture. Although the ACSW modifier was detrimental to the low-temperature failure tensile strain, an appropriate amount of WCO could compensate for the lack of low-temperature properties in the ACSW single-modified asphalt mixture because the polar groups in WCO and asphalt became attracted to each other, and WCO is mainly composed of small molecules with desirable fluidity, which reduces the intermolecular forces in the modified asphalt. Therefore, the introduction of WCO significantly improved the low-temperature anti-cracking. Comparing the ITS and failure tensile strain of asphalt mixtures, it is discovered that the 6%ACSW and 2%WCO compound modification performed best in terms of deformation ability and being able to withstand higher loads at low temperatures.

#### 3.2.2. Tensile Performance

**Figure 7:**
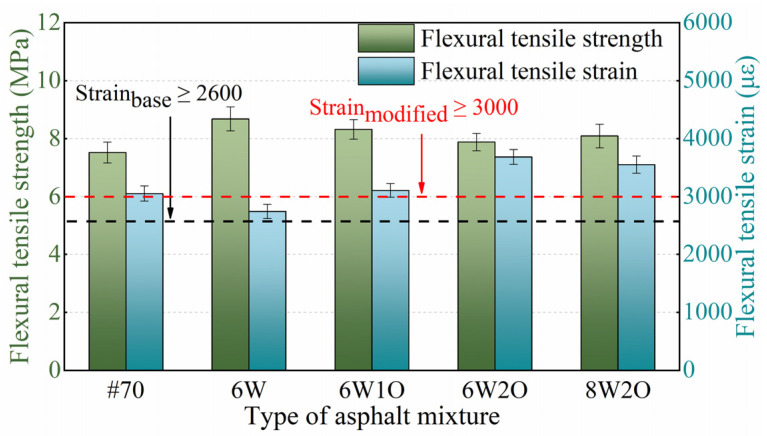
Test results of low-temperature flexural tensile strength and stain.

Flexural tensile strength and maximum flexural tensile strain are indicators used to evaluate the low-temperature tensile properties of asphalt mixtures [44]. Greater flexural tensile strength and greater maximum flexural tensile strain indicate that the asphalt mixture has a more desirable low-temperature tensile performance [45]. The three-point bending test results are presented in Figure 7. The low-temperature maximum flexural tensile strain of the base asphalt mixture was 3052 με, which meets the requirement of greater than 2600 με in cold regions with severe winters [39]. In addition, except for the ACSW single-modified asphalt mixture, the maximum flexural tensile strain of the other compound-modified asphalt mixtures met the standards of the modified asphalt mixture no less than 3000 με [39]. The maximum flexural tensile strain of the 6%ACSW modified asphalt mixture decreased, indicating that the single modification of ACSW is detrimental to low-temperature properties. After adding WCO, the flexural tensile strain of the modified asphalt mixture slightly increased. When the WCO content continued to increase to 2%, the flexural tensile strain was enhanced by 34.2% and 20.7%, respectively, compared to the ACSW single-modified and base asphalt mixtures. On this basis, when the ACSW modifier was increased to 8%, the flexural tensile strain of the 8%ACSW and 2%WCO compound-modified asphalt mixture was slightly reduced but still obviously greater than that of the ACSW single-modified and base asphalt mixtures. It shows that the WCO offered an outstanding contribution to improving the asphalt mixture’s low-temperature tensile performance. This phenomenon could be that adding WCO improves the flexibility of the asphalt mixture by reference to the paper by Niu et al. [35], thus enhancing the resistance to cracking to a certain degree at low temperatures. Based on a comprehensive comparison of four different ratios of modified asphalt mixtures, the 6%ACSW and 2%WCO compound-modified asphalt mixture had greater flexural tensile strength, as well as a more desirable maximum flexural tensile strain at low temperatures.

### 3.3. Moisture Susceptibility

#### 3.3.1. Marshall Stability after Immersion

**Figure 8:**
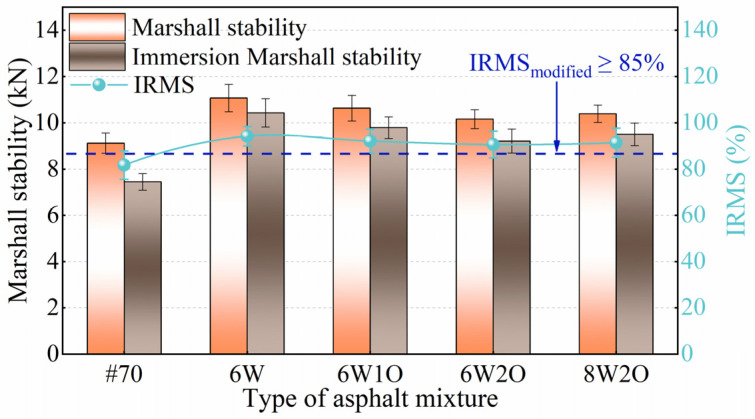
Test results of immersion Marshall stability.

Water damage is one of the most significant factors that weaken the binding strength of asphalt binders and aggregates, resulting in severe spalling and potholes in asphalt pavement. IRMS was employed to investigate the moisture susceptibility of the modified asphalt mixture at high temperatures. As exhibited in Figure 8, the IRMS of all types of modified asphalt mixtures fulfilled the specification requirement of 85% or higher [39]. The IRMS of the 6%ACSW modified asphalt mixture was 94.2%, representing a 12.5% increase over the base asphalt mixture because the organic–inorganic surface co-treatment of whiskers improved the bonding between the asphalt binder and aggregate, making the modified asphalt mixture less susceptible to the effects of water. It is found that after introducing WCO, the IRMS of the compound-modified asphalt mixtures were all slightly less than those of the ACSW single-modified asphalt mixture. The content of WCO also influenced the IRMS of the asphalt mixture. The IRMS reduced with an increase in WCO content and grew along with the ACSW content. However, the IRMS of the compound-modified asphalt with ACSW and WCO in appropriate proportions increased to variable levels compared to the base asphalt mixture. The IRMS of the 6%ACSW and 1%WCO, 6%ACSW and 2%WCO, 8%ACSW and 2%WCO compound-modified asphalt mixtures were 92.1%, 90.6%, and 91.4%, respectively, representing increases of 10.4%, 8.9%, and 9.7% compared to the base asphalt mixture.

#### 3.3.2. Indirect Tensile Strength after Freeze–Thaw Damage

**Figure 9:**
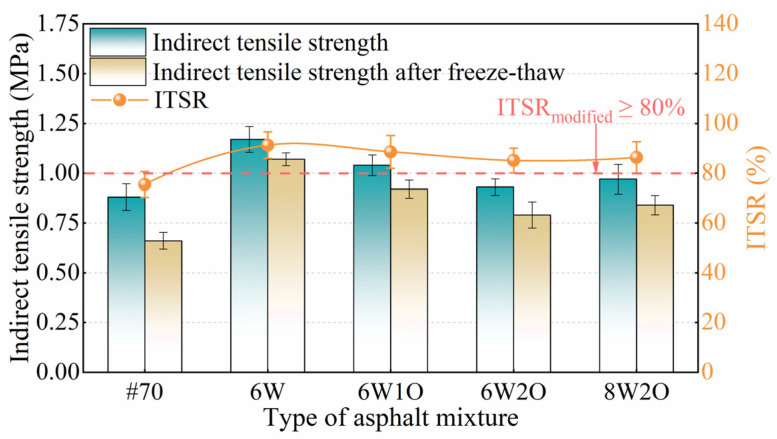
Test results of indirect tensile strength after freeze–thaw.

The low-temperature moisture susceptibility results are presented in Figure 9. According to the requirements of the technical specifications [39], the ITSR of the base asphalt mixture and modified asphalt mixture ought to be more than 75% and 80%, respectively, in areas where the average rainfall exceeds 500 mm. Notably, all types of asphalt mixtures fulfill the relevant standards. In addition, the higher the ITSR of the modified asphalt mixture is, the better the mixture’s resistance to water damage. The ITSR and IRMS of the asphalt mixtures showed similar trends. The ITSR of the 6%ACSW modified asphalt mixture was maximum with 91.3%, which was 15.8% greater than that of the base asphalt mixture, again proving that ACSW can improve moisture susceptibility. The ITSR of the ACSW and WCO compound-modified asphalt mixtures were slightly lower than those of the single-modified asphalt mixture. The ITSR of the 6%ACSW and 1%WCO, 6%ACSW and 2%WCO, and 8%ACSW and 2%WCO compound-modified asphalt mixtures increased by 13.1%, 9.6%, and 10.8%, respectively, compared to the base asphalt mixture. This result indicates that adding the appropriate proportions of ACSW and WCO to the base asphalt guarantee desirable moisture sensitivity of the asphalt mixture.

### 3.4. Fatigue Performance

**Figure 10:**
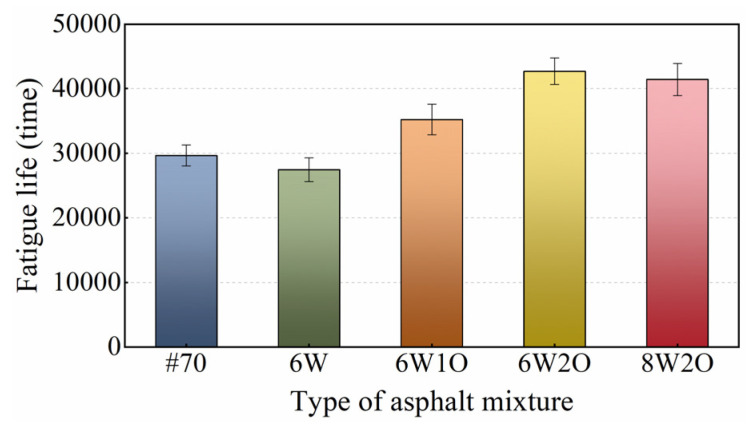
Test results of fatigue performance.

The four-point bending test results are presented in Figure 10. Compared to the base asphalt mixture, the fatigue life of the ACSW modified asphalt mixture was slightly enhanced, while the fatigue life of the compound-modified asphalt mixtures increased obviously. Among them, the 6%ACSW and 2%WCO compound-modified asphalt mixture offered the best fatigue life, followed by that of the 8%ACSW and 2%WCO, 6%ACSW and 1%WCO compound-modified asphalt mixtures. Compared with the base asphalt mixture, the fatigue life of the 6%ACSW and 2%WCO compound-modified asphalt mixture increased by 44%, and the fatigue performance was enhanced notably. The fatigue life of the asphalt mixture was enhanced with an increase in WCO content or a decrease in ACSW content, indicating that WCO had a positive impact on fatigue life, while ACSW had a negative influence. However, the fatigue lives of the above compound-modified asphalt mixtures were still superior to those of the base asphalt mixture, demonstrating that WCO was able to compensate for the fatigue life loss caused by ACSW. After ACSW and WCO compound modification, the asphalt mixture achieved a higher fatigue life, improving the life span of the asphalt pavement.

### 3.5. Anti-Freezing Performance

**Figure 11:**
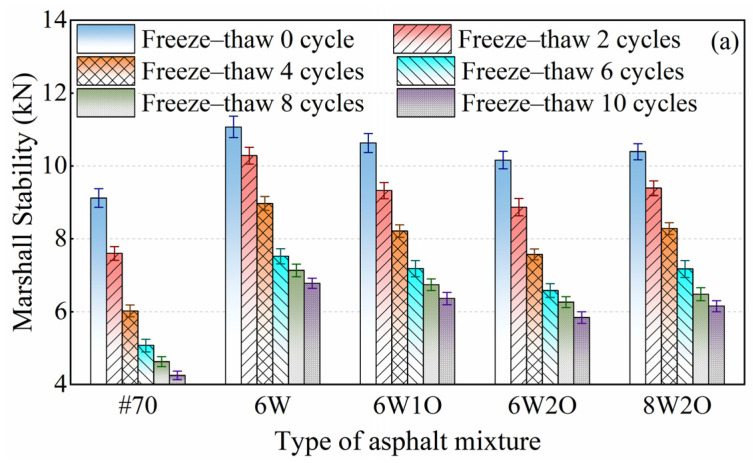

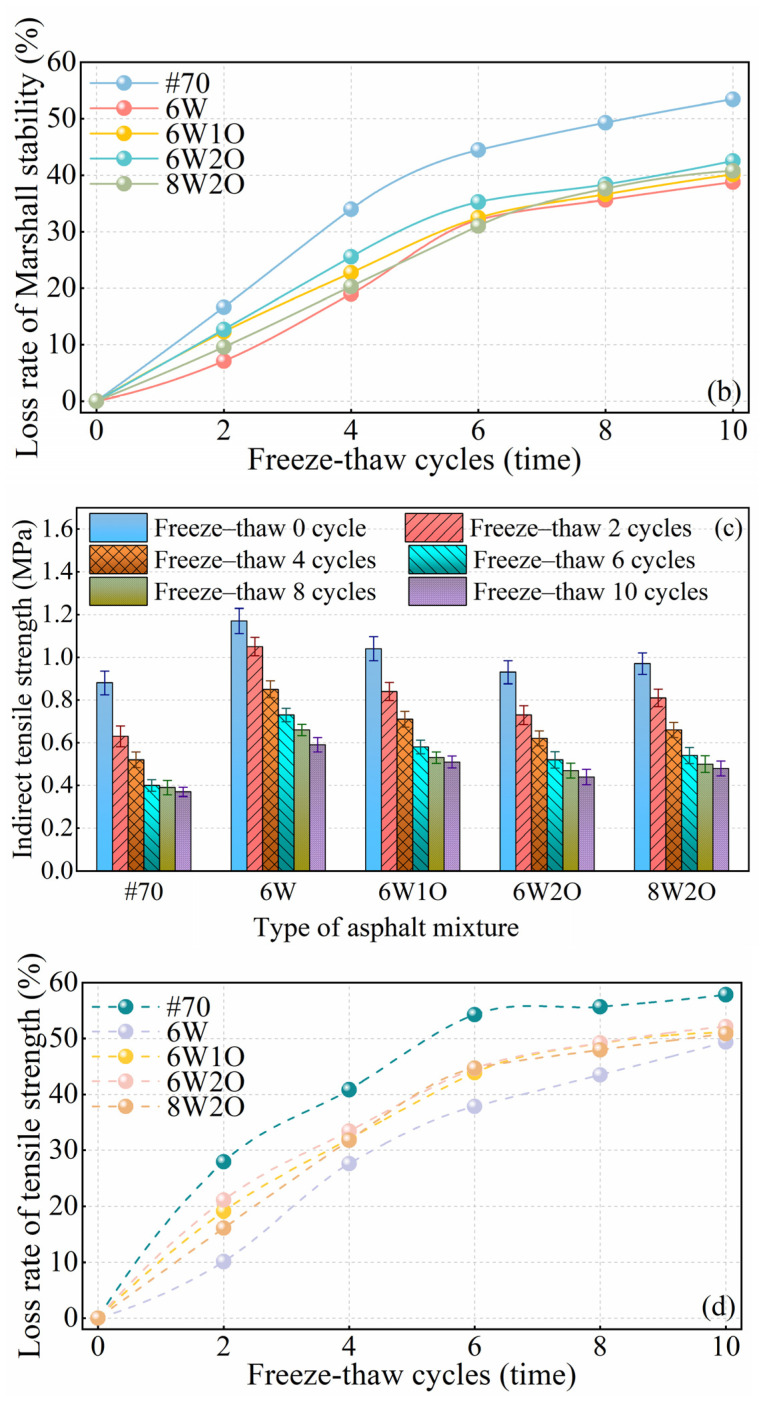
Test results of anti-freezing performance. (**a**) Test results of Marshall Stability after repeated freeze–thaw cycles. (**b**) Test results of loss rate of Marshall Stability after repeated freeze–thaw cycles. (**c**) Test results of indirect tensile strength after repeated freeze–thaw cycles. (**d**) Test results of loss rate of tensile strength after repeated freeze–thaw cycles.

The repeated freeze–thaw cycle test is analogous to the moisture susceptibility test, but it provides a more complete representation of the damage caused to the asphalt mixture through the cyclic repetitive action of water freezing and erosion. The changes in the MS and ITS of asphalt mixtures after repeated freeze–thaw cycles are illustrated in Figure 11. The MS and ITS of all asphalt mixtures continually decreased, while the loss rates increased, under repeated freeze–thaw cycles. At the initiation of the freeze–thaw cycle, the MS and ITS decreased quickly. After six repeated freeze–thaw cycles, the loss rate of stability and strength slowed down. At the initial stage of the freeze–thaw cycle, the internal voids of the asphalt mixture specimens became enlarged under the effect of water freezing. External water was able to invade the internal voids and the asphalt binder film above 0 °C, resulting in a significant weakening of the asphalt mixture’s performance. By increasing the number of repeated freeze–thaw cycles, the internal voids of the asphalt mixture connected to form channels, which were able to release the pressure formed by water freezing, and the freeze–thaw damage was slowed down at this time.

The MS (Figure 11a) and ITS (Figure 11c) of the modified asphalt mixtures were still higher, while the loss rates of MS (Figure 11b) and ITS (Figure 11d) were notably below those of the base asphalt mixture. The loss rates of MS and ITS of ACSW modified asphalt mixture were 14.7% and 8.5% less than those of the base asphalt mixture, which indicated that ACSW could substantially enhance the anti-freezing performance. After introducing WCO, the loss rate increased slightly. However, the loss rates of the MS and ITS of the compound-modified asphalt mixtures were also below the base rates. For example, the loss rates of MS and ITS of the 6%ACSW and 2%WCO compound-modified asphalt mixture were 10.9% and 5.7% less than those of the #70 asphalt mixture. Therefore, the ACSW and WCO compound modification method was effective in promoting anti-freezing performance and could be applied to frozen areas.

### 3.6. Anti-Aging Performance

**Figure 12:**
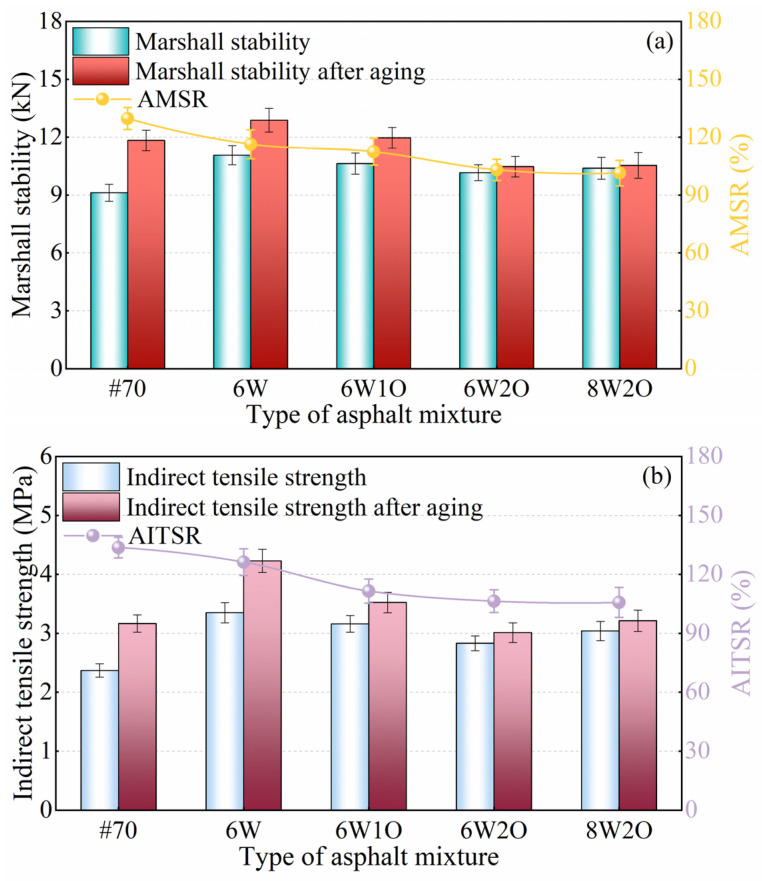
Test results of anti-aging performance. (**a**) Test results of Marshall stability and AMSR after aging. (**b**) Test results of indirect tensile strength and AITSR after aging.

Oxidation aging causes hardening of the asphalt, which reduces the performance of the asphalt mixture. AMSR and AITSR were used to characterize the anti-aging properties of the modified asphalt mixtures, and the test results are illustrated in Figure 12. Compared to the unaged asphalt mixtures, the MS increased after aging (Figure 12a). This result may be due to the evaporation of the light component of the asphalt binder and the conversion of the saturated and aromatic components into high molecular weight asphaltene and resins, thus increasing the viscosity of the asphalt, a harder and more brittle asphalt mixture. In general, the asphalt mixture had ideal aging resistance when the AMSR increased to 100%. The AMSR of the ACSW modified asphalt mixture was reduced by 13.4% compared to that of the base asphalt mixture, indicating that the aging resistance of the asphalt mixture was enhanced by ACSW modification. After ACSW and WCO compound modification, the AMSR of the asphalt mixture was further reduced compared to that of the ACSW single-modified asphalt mixture, gradually approaching 100%. For example, the AMSR of the 6%ACSW and 2%WCO asphalt mixture was 103.1%. The higher the amount of WCO, the greater the high-temperature aging resistance of the asphalt mixture. This result is possible because the inorganic–organic surface co-treatment of ACSW can cross-link the internal components of the asphalt to form a tight network, while WCO can provide the light components missing in the aging process. Thus, ACSW and WCO compound modification was found to have an active anti-aging effect at high temperatures.

The low-temperature test results after aging are shown in Figure 12b. Here, the trend between AITSR and AMSR is similar to the change in modifier type and content. The ITS of asphalt mixtures were enhanced to some degree because the aged asphalt mixtures became stiff and brittle, thereby improving tensile strength at low temperatures. The AITSR of the ACSW asphalt mixture decreased by 7.4% compared to that of the base asphalt mixture, indicating that the low-temperature anti-aging of the asphalt mixture improved after ACSW modification. After adding ACSW and WCO, the AITSR of the asphalt mixture continued to reduce compared to that of the ACSW single-modified mixture. For example, the ITSR of the 6%ACSW and 2%WCO compound-modified asphalt mixture was 106.4%, which tended to be 100%, indicating that this mixture’s low-temperature performance was almost unaffected by aging. In addition, the AITSR of all compound-modified asphalt mixtures were below those of the base asphalt mixture, indicating that ACSW and WCO compound modification improved the anti-aging effects of the asphalt mixture at low temperatures.

## 4. Conclusions

This study investigated the feasibility of using anhydrous calcium sulfate whiskers (ACSW) and waste cooking oil (WCO), two types of recycled waste, to improve the properties of compound-modified asphalt mixtures. The goal was to boost the natural asphalt quality, as well as eliminate the environmental pollution caused by phosphogypsum and WCO. A series of performance evaluation tests of the asphalt mixtures were performed to validate the compound-modification efficiency. The primary findings are as follows:

(1) The dynamic stability of the modified asphalt mixture increases by 97.3% after incorporating 6%ACSW, compared to the base asphalt mixture. ACSW enhanced the high-temperature performance of the base asphalt mixture, which substantially enhanced the anti-rutting resistance of the asphalt mixture at high temperatures.
(2) The flexural tensile strain of the compound-modified asphalt mixture increases by 34.2% and 20.7% after adding 2%WCO, compared to the ACSW modified and base asphalt mixtures. WCO had a beneficial effect on the low-temperature performance of the asphalt mixture. WCO compensated for the dramatic reduction in the low-temperature tensile strain of the ACSW single-modified asphalt mixture.
(3) The indirect tensile strength residual ratio after the freeze–thaw, and fatigue life of the 6%ACSW and 2%WCO compound-modified asphalt mixture increased by 9.6% and 44%, respectively, compared to the base asphalt mixture. The moisture susceptibility and fatigue performance of the asphalt mixture were improved after ACSW and WCO compound modification.
(4) The Marshall stability and indirect tensile strength ratio loss rate of 6%ACSW and 2%WCO compound-modified asphalt mixture after repeated freeze-thaw cycles were 10.9% and 5.7% lower than those of the base asphalt mixture. The aging Marshall stability ratio and aging indirect tensile strength ratio of all compound-modified asphalt mixtures were below those of the base asphalt mixture. After repeated freeze–thaw cycles and long-term aging, the ACSW and WCO compound-modified asphalt mixtures changed less than that of the base asphalt mixture.
(5) Adding an appropriate amount of ACSW and WCO is a feasible way to improve asphalt mixture performance. Comprehensively considering the high- and low-temperature performance, moisture susceptibility, fatigue, anti-freezing, and anti-aging properties, we found 6%ACSW and 2%WCO compound modification to be the best option to improve asphalt mixture performance.

## Figures and Tables

**Table 1:** Physical parameters of base asphalt.

Property	Unit	Result	Specification Requirement [39]
Penetration (25 °C)	0.1 mm	75.7	60–80
Ductility (10 °C)	cm	23.8	≥15
Softening point	°C	47.1	≥45
Penetration index	/	−0.62	−1.5~1.0
Rotational viscosity (135 °C)	Pa·s	0.307	/
RTFOT Quality loss (163 °C)	%	0.27	<±0.8
RTFOT Residual penetration ratio (25 °C)	%	67.6	>61
163 °C RTFOT Residual ductility (10 °C)	cm	8.9	>6

**Table 2:** Physical properties of ACSW.

Project	Unit	Physical Property
Appearance	/	White flocculent powder
Whiteness	%	≥92
Diameter	μm	1–4
Length	μm	10–30
Density	g·cm^−3^	2.69
Loose density	g·cm^−3^	0.3–0.65
Extension strength	GPa	20.5
Elasticity modulus	GPa	178
Melting point	°C	1450
Heat resistance	°C	1000
PH	/	7 ± 0.5

**Table 3:** Physical properties of WCO.

Property	Unit	Value
Acid value	mg KOH/gm	3.55
Density (15 °C)	gm/cm^3^	0.91
Viscosity (25 °C)	Pa·s	0.22
Moisture content	%	0.34
Appearance	-	Brown-yellow greasy liquid

**Table 4:** Physical parameters of aggregates and mineral filler.

Index		Test Result		Specification Requirement [39]
		5–10 mm	10–15 mm	
Coarse aggregate	Crushed value/%	/	12.5	≤26
	Wear loss rate/%	10.2	10.7	≤28
	Apparent relative density	3.11	3.08	≥2.6
	Water absorption rate/%	1.13	0.56	≤2
	Ruggedness/%	4	4	≤12
	Needle and flake particles content/%	7.5	6.3	≤15
	<0.075 mm particles content/%	0.8	0.5	≤1
	Soft stone content/%	1.8	1.9	≤3
	Adhesion	/	Level 4	≥Level 4
	Polished stone value	/	47	≥42
Fine aggregate	Apparent relative density	2.61		≥2.5
	Ruggedness/%	3.7		≤12
	Mud content (<0.075 mm)	1.9		≤3
	Sand equivalent	74.8		≥60
Mineral filler	Apparent density/t/m^3^	2.73		≥2.50
	Water content/%	0.21		≤1
	Hydrophilic coefficient	0.77		<1
	Plasticity index/%	2.6		<4
	Appearance	No agglomeration		No agglomeration

**Table 5:** The abbreviations and physical properties of different types of asphalt binders.

Asphalt Binder Type	Abbreviation	Penetration/0.1 mm	Ductility (10 °C)/cm	Softening Point/°C	Viscosity (135 °C)/Pa·s
Base asphalt	#70	75.7	23.8	47.1	0.307
6%ACSW modified asphalt	6W	50.3	6.6	57.4	0.659
6%ACSW and 1%WCO compound-modified asphalt	6W1O	54.9	11.7	56.1	0.588
6%ACSW and 2%WCO compound-modified asphalt	6W2O	60.9	23.1	54.6	0.534
8%ACSW and 2%WCO compound-modified asphalt	8W2O	59	19	55.8	0.578

**Table 6:** Results of OAC and the corresponding parameters.

Asphalt Mixture Type	#70	6W	6W1O	6W2O	8W2O
OAC/%	4.24	4.58	4.45	4.33	4.41
Bulk volume relative density	2.463	2.414	2.392	2.371	2.388
Volume of air voids (VV)/%	4.53	4.16	4.21	4.24	4.18
Voids in mineral aggregate (VMA)/%	15.31	14.63	14.87	15.16	15.07
Voids filled with asphalt (VFA)/%	70.41	71.57	71.69	72.03	72.26
Marshall stability/kN	9.12	11.07	10.63	10.16	10.39
Flow value/mm	3.34	2.76	2.96	3.12	3.08

## Data Availability

All data that support the findings of this study are included within the article.
